# Revenue Generation and Follow-up for a Hand Trauma Program for Emergency Department Patients in an Inner-City Metropolitan Area

**DOI:** 10.5435/JAAOSGlobal-D-23-00173

**Published:** 2023-11-17

**Authors:** Stephanie Nulty, Jessi Fore, John Madison, Charles S. Day

**Affiliations:** From Henry Ford Health, Detroit, MI.

## Abstract

**Background:**

Although hand trauma care has proved to be profitable, loss of trauma patients from a system may lead to revenue loss. Our study aimed to (1) elucidate the economic effect of hand trauma programs, (2) quantify the potential fiscal effect of loss of follow-up, and (3) determine factors contributing to leakage of patients from the healthcare system.

**Methods:**

Revenue data were retrospectively extracted for all adult hand trauma patients within a multicenter healthcare system from 2014 to 2018. Demographic and encounter factors were analyzed using Wilcoxon rank-sum test for differences in continuous variables, Pearson chi square test for categorical variables, and odds ratios. A follow-up model was created using logistic regression.

**Results:**

A total of 56,995 (31% new, 69% established) hand trauma encounters were recorded. Follow-up was markedly affected by many factors, including new vs. established patients. Of the 17,748 new patients, 8638 (48.6%) returned for subsequent care, generating $34M. The patients who did not return may have lost $176M for the system.

**Conclusions:**

Many factors lead to loss of follow-up. Understanding these factors can help target efforts to minimize leakage of hand trauma patients. Hand trauma introduces new patients to hospitals, generating notable revenue. Leakage of hand trauma patients has substantial revenue losses.

The cost of health care in the United States is continuing to rise, and by 2025, it is estimated to account for 19.9% of the gross domestic product.^1^ One of the largest portions of the healthcare industry is dedicated to caring for musculoskeletal disorders.^2^ As a result, there is considerable revenue generated from this sector of health care.^3,4^ Fracture care alone accounts for nearly $100 billion per year, with even more costs coming from other traumatic orthopaedic injuries.^5,6^ Specifically, hand trauma accounts for more than 12% of all trauma cases; within this 12%, the most prevalent injuries are hand/wrist fractures that occur in 179 of 100,000 trauma patients.^7^ With this high volume, hand trauma has the potential to create a notable stream of revenue for a hospital system.

While hand trauma programs have previously been fiscally advantageous to hospital systems,^8^ hand surgery service lines (not exclusively hand trauma) have demonstrated notable revenue generation for hospital systems because of the prevalence of hand surgery cases and the follow-up opportunities they create.^6,8^ Studies have been conducted to analyze the continued revenue generated by subsequent visits after orthopaedic trauma presentation.^9^ Despite the demonstrated financial advantage of orthopaedic trauma and the known volume of hand trauma, the financial benefit of a hand trauma program has not been studied directly.

Many patients presenting with traumatic injuries are new patients who have yet to have an interaction with the healthcare system. A unique opportunity is presented for a patient to establish themselves in the healthcare system after trauma presentation. It is not well-understood whether patients will continue to follow-up with the healthcare system that they initially presented to. Leakage of trauma patients to follow-up may be a challenge within many healthcare specialties, and it can account for a notable loss in revenue and loss of continuity of care for patients.^9^ Therefore, it is crucial for a healthcare system to be able to identify the patient factors that contribute to a decreased likelihood of following up within the system.

Currently, there is not yet a strong understanding of the patient factors that contribute to loss of follow-up and the downstream financial effect of this forfeiture of revenue. Therefore, the primary purpose of this study was to elucidate the economic effect of hand trauma care and patient loss to follow-up across the orthopaedic hand trauma service for injury/noninjury-related services after initial hand trauma presentation. The secondary purpose was to determine the patient factors that contributed to increasing the likelihood of follow-up within the healthcare system after trauma. Our primary hypothesis is that older, established male patients are more likely to follow up. Our secondary hypothesis is that the greatest revenue would be from rehabilitation services.

## Methods

Data were retrospectively collected over 4 years, from January 1, 2014, to December 31, 2018, from a large integrated multicenter academic healthcare system in the Midwest. The population included adult orthopaedic patients presenting for hand trauma evaluation. Any encounter with an ICD-10 code for adult hand trauma presenting to the emergency department was included in this study. All patients were provided with written follow-up care instructions on discharge, and a standardized discussion of follow-up was included in discharge. Interpreters were available at all times and were used during all discussions for any patient with difficulty comprehending English. Follow-up within 6 weeks of hand trauma presentation was recorded. Injury-related care was defined as subsequent care for a hand ICD-10 code. We followed patients for 2 years after the initial presentation to record all subsequent care.

Demographic information including age, sex, race, ethnicity, insurance type (Medicaid Health Maintenance Organization (HMO), Blue Shield or commercial, HAP, Medicaid, Medicare, Medicare Advantage, self-pay, Tricare, and worker's compensation), new vs. established patient status, outside-hospital transfer, hospital admission, intensive care unit admission, hand surgery consultation, and trauma designation of center presented to were analyzed. For patient status, patients whose first interaction with our healthcare system occurred at the presentation of their hand trauma were considered new patients. Established patient status refers to patients who had an interaction with our healthcare system before their hand trauma presentation.

Financial data collected included revenue, cost, and operating margin related to hand trauma presentation and subsequent care. This included both inpatient and outpatient care. Injury-related and injury-unrelated services were included in the analysis. Follow-up was within 24 months after the initial trauma presentation. Lost revenue was calculated by determining the average revenue generated per patient and using that average to extrapolate the potential loss from each patient lost to follow-up.

After these data were collected, Wilcoxon rank-sum test was used to test for differences in continuous variables, Pearson chi square test for categorical variables, and odds ratio for each variable. A follow-up model was created using logistic regression. Results with a *P* value of < 0.05 were considered statistically significant.

## Results

### Demographics

We included 56,995 patients; most of them were White (56%) and male (54%) (Table 1). The average age was 38 years. There were more established patients (69%) than new patients (31%). A small proportion of patients received formal hand consultation (4.3%), were admitted to the intensive care unit (0.1%) or hospital (2.3%), or were transferred from an outside system (2.7%). The most common insurance provider was Medicaid HMO (33%).

**Table 1:** Characteristics of 56,995 Patients

Variable	N = 56,995^a^
Sex	
Male	30,990 (54%)
Female	26,004 (46%)
NULL	1
Age	38 (22)
	3
Race	
White	31,683 (56%)
Black	18,555 (33%)
Other	6756 (12%)
NULL	1
Established status	
Active	39,247 (69%)
New	17,748 (31%)
External Referral	1527.000 (2.679%)
Hospital admission	1303 (2.3%)
ICU Admission	70 (0.1%)
Follow-up	22,689 (40%)
Ethnic group	
Not Hispanic, Arab or Chaldean	51,083 (90%)
Arab or Chaldean	1561 (2.7%)
Decline/Not known	1947 (3.4%)
Hispanic or Latino	2389 (4.2%)
NULL	15
Consult Flag	2429 (4.3%)
Insurance	
Medicaid HMO	19,085 (33%)
Blue Shield	10,504 (18%)
Commercial	3886 (6.8%)
HAP	3528 (6.2%)
Medicaid	3614 (6.3%)
Medicare	5741 (10%)
Medicare Advantage	3926 (6.9%)
Self-pay	3973 (7.0%)
Tricare	203 (0.4%)
Worker's comp	2535 (4.4%)
Level of trauma	
Level I	10,259 (18%)
Level II	11,380 (20%)
Level III	12,736 (22%)
No trauma designation	22,620 (40%)

HMO = Health Maintenance Organization.

a n (%); mean (SD).

### Return for Follow-Up

We observed a 39.8% follow-up rate for any care within 6 weeks (22,689 of 56,995) and a 68% return for care within 2 years (38,720 of 56,995) (Table 2). 15.9% of patients returned specifically for subsequent care related to hand trauma.

**Table 2:** All Patients Return for Follow-Up With Odds Ratios

Variable	N	Follow-up					
		No, N = 34,306^a^	Yes, N = 22,689	*P* ^b^	OR	95% CI	*P*
Sex	56,994			<0.001			
Male		19,110 (56%)	11,880 (52%)		—	—	
Female		15,195 (44%)	10,809 (48%)		0.99	0.96, 1.03	0.7
Age	56,992	34 (20)	43 (22)	<0.001	1.01	1.01, 1.02	<0.001
Race	56,994			<0.001			
White		18,545 (54%)	13,138 (58%)		—	—	
Black		11,201 (33%)	7354 (32%)		1.01	0.96, 1.05	0.8
Other		4559 (13%)	2197 (9.7%)		0.9	0.84, 0.96	0.001
Established status	56,995			<0.001			
Active		21,334 (62%)	17,913 (79%)		—	—	
New		12,972 (38%)	4776 (21%)		0.5	0.48, 0.52	<0.001
External referral	56,995	731 (2.1%)	796 (3.5%)	<0.001	1.48	1.40, 1.82	<0.001
Hospital admission	56,995	382 (1.1%)	921 (4.1%)	<0.001	1.59	1.40, 1.82	<0.001
ICU admission	56,995	15 (<0.1%)	55 (0.2%)	<0.001	2.08	1.14, 4.02	0.022
Ethnic group	56,980			<0.001	—	—	
Not Hispanic, Arab or Chaldean		30,445 (89%)	20,638 (91%)		—	—	
Arab or Chaldean		1183 (3.4%)	378 (1.7%)		0.67	0.59,0.76	<0.001
Decline/Not known		1255 (3.7%)	692 (3.0%)		0.87	0.79, 0.97	0.009
Hispanic or Latino		1408 (4.1%)	981 (4.3%)		1.26	1.15, 1.38	<0.001
Consult Flag	56,995	787 (2.3%)	1642 (7.2%)	<0.001	2.85	2.59, 3.15	<0.001
Insurance	56,995			<0.001			
Commercial/Blue Cross		11,909 (35%)	6962 (31%)		—	—	
HAP		1757 (5.1%)	1795 (7.9%)		1.59	1.47, 1.71	<0.001
Medicaid/Medicaid HMO		12,522 (37%)	6168 (27%)		0.86	0.83, 0.90	<0.001
Medicare/Medicare Advantage		3840 (11%)	5816 (26%)		1.35	1.27, 1.44	<0.001
Self/Uninsured		2828 (8.2%)	829 (3.7%)		0.55	0.51, 0.60	<0.001
Worker's comp		1449 (4.2%)	1119 (4.9%)		1.19	1.09, 1.30	
Level of trauma	56,995						
Level I		5872 (17%)	4387 (19%)		—	—	
Level II		6173 (18%)	5207 (23%)		1.23	1.15, 1.31	<0.001
Level III		7534 (22%)	5202 (23%)		0.98	0.92, 1.04	0.5
No trauma designation		14,727 (43%)	7893 (35%)	<0.001	0.88	0.83, 0.93	<0.001

CI = confidence interval, HMO = Health Maintenance Organization, OR = odds ratio.

a n (%); mean (SD).

b Pearson chi square test; Wilcoxon rank-sum test.

For new and established patients (Table 3) returning for hand-related follow-up, there were two patient demographic factors that led to a greater likelihood of follow-up: increasing age (OR 1.01 per year of age, *P* < 0.001) and ‘Hispanic or Latino’ (OR 1.25, *P* < 0.001). Two visit-specific factors that conferred greater follow-up were transfer from an outside system (OR 1.73, *P* < 0.001) and hand surgery consultation (OR 3.66, *P* < 0.001). Certain payment factors (worker’s comp [OR 1.19, *P* < 0.001], HAP [OR 1.59, *P* < 0.001]) resulted in increased follow-up.

**Table 3:** All Patients Return for Hand Follow-Up With Odds Ratios

Variable	N	Hand Follow-up					
		No, N = 47,927^a^	Yes, N = 9,068^a^	*P* ^b^	OR	95% CI	*P*
Sex	56,994			<0.001			
Male		25,657 (54%)	5333 (59%)		—	—	
Female		22,269 (46%)	3735 (41%)		0.77	0.73, 0.80	<0.001
Age	56,992	37 (21)	42 (22)	<0.001	1.01	1.01, 1.01	<0.001
Race	56,994			<0.001			
White		26,435 (55%)	5248 (58%)		—	—	
Black		15,661 (33%)	2894 (32%)		0.95	0.90, 1.01	0.094
Other		5830 (12%)	926 (10%)		0.91	0.84, 0.99	0.029
Established status	56,995			<0.001			
Active		32,437 (68%)	6810 (75%)		—	—	
New		15,490 (32%)	2258 (25%)		0.7	0.66, 0.74	<0.001
External transfer	56,995	1099 (2.3%)	428 (4.7%)	<0.001	1.73	1.53, 1.95	<0.001
Hospital admission	56,995	946 (2.0%)	357 (3.9%)	<0.001	0.97	0.84, 1.12	0.7
ICU admission	56,995	57 (0.1%)	13 (0.1%)	0.5	0.62	0.31, 1.13	0.14
Insurance type	56,994			<0.001			
Commercial/Blue Cross		15,834 (33%)	3037 (33%)		—	—	
HAP		2732 (5.7%)	820 (9.0%)		1.49	1.37, 1.63	<0.001
Medicaid/Medicaid HMO		16,393 (34%)	2297 (25%)		0.72	0.68, 0.77	<0.001
Medicare/Medicare Advantage		7734 (16%)	1922 (21%)		0.91	0.84, 0.98	0.02
Self/Uninsured		3319 (6.9%)	338 (3.7%)		0.53	0.47, 0.60	<0.001
Worker's comp		1914 (4.0%)	654 (7.2%)		1.61	1.46, 1.78	<0.001
Hand follow-up	56,995	13,621 (28%)	9068 (100%)	<0.001			
Ethnic group	56,980			<0.001			
Not Hispanic, Arab or Chaldean		42,884 (90%)	8199 (90%)		—	—	
Arab or Chaldean		1435 (3.0%)	126 (1.4%)		0.63	0.52, 0.75	<0.001
Decline/Not known		1626 (3.4%)	321 (3.5%)		1.05	0.92, 1.19	0.5
Hispanic or Latino		1967 (4.1%)	422 (4.7%)		1.25	1.11, 4.04	<0.001
ED Location	56,995			<0.001	—	—	
Henry Ford Main		8286 (17%)	1973 (22%)				
Henry Ford Allegiance		3028 (6.3%)	908 (10%)				
Henry Ford Cottage		1656 (3.5%)	363 (4.0%)				
Henry Ford Fairlane		9116 (19%)	1396 (15%)				
Henry Ford Sterling Heights		3119 (6.5%)	539 (5.9%)				
Henry Ford Macomb		6222 (13%)	1222 (13%)				
Henry Ford West Bloomfield		3961 (8.3%)	738 (8.1%)				
Henry Ford Wyandotte		6951 (15%)	1086 (12%)				
Henry Ford Brownstown		5588 (12%)	843 (9.3%)				
Consult Flag	56,995	1429 (3.0%)	1000 (11%)	<0.001	3.66	3.32, 4.04	<0.001
Location-based level of trauma	56,995			<0.001			
Level I		8286 (17%)	1973 (22%)		—	—	
Level II		9250 (19%)	2130 (23%)		1.1	1.01, 1.19	0.032
Level III		10,912 (23%)	1824 (20%)		0.84	0.77, 0.91	<0.001
No trauma designation		19,479 (41%)	3141 (35%)		0.89	0.83, 0.96	0.003

CI = confidence interval, HMO = Health Maintenance Organization, OR = odds ratio.

a n (%); mean (SD).

b Pearson chi square test; Wilcoxon rank-sum test.

Decreased likelihood of hand trauma–related follow-up for new and established patients was seen with female (OR 0.77, *P* < 0.001), ‘other’ race (OR 0.91, *P* = 0.029), Medicaid/Medicaid HMO (OR 0.86, *P* < 0.001), Medicare/MA (OR 0.91, *P* = 0.02), and self-pay (OR 0.55, *P* < 0.001) patients.

When examining only new patients returning for subsequent hand trauma–related care, visit factors leading to increased follow-up included hospital admission, external transfer, and hand surgery consultation (OR 1.48, *P* = 0.009, OR 1.94, *P* < 0.001, and OR 4.06, *P* < 0.001, respectively). Similar visit factors were associated with increased follow-up in new patients returning for hand trauma–unrelated care (Table 4). Payment factors leading to increased follow-up included only patients using worker's compensation (OR 1.76, *P* < 0001).

**Table 4:** Analysis of New Patients Returning for Injury-Unrelated Follow-Up With Odds Ratios

Variable	N	Follow-up					
		No, N = 12,972^a^	Yes, N = 4,776^a^	*P* ^b^	OR^c^	95% CI^d^	*P*
Sex	17,747			<0.001			
Male		8035 (62%)	3145 (66%)		—	—	
Female		4936 (38%)	1631 (34%)		0.86	0.80, 0.92	<0.001
Age	17,747	32 (19)	36 (20)	<0.001	1.01	1.01, 1.01	<0.001
Race	17,747			0.003			
White		7384 (57%)	2740 (57%)		—	—	
Black		3637 (28%)	1409 (30%)		1.08	0.99, 1.19	0.085
Other		1950 (15%)	627 (13%)		0.98	0.87, 1.09	0.7
Established status	17,748			>0.9			
Active		0 (0%)	0 (0%)		—	—	
New		12,972 (100%)	4776 (100%)				
External referral	17,748	259 (2.0%)	200 (4.2%)	<0.001	1.76	1.43, 2.15	<0.001
Hospital admission	17,748	96 (0.7%)	191 (4.0%)	<0.001	2.35	1.78, 3.10	<0.001
ICU admission	17,748	6 (<0.1%)	22 (0.5%)	<0.001	2.53	0.96, 7.97	0.079
Ethnic group	17,734			<0.001			
Not Hispanic, Arab or Chaldean		11,429 (88%)	4242 (89%)		—	—	
Arab or Chaldean		512 (4.0%)	126 (2.6%)		0.88	0.71, 1.08	0.2
Decline/Not known		461 (3.6%)	143 (3.0%)		0.97	0.79, 1.18	0.8
Hispanic or Latino		556 (4.3%)	265 (5.5%)		1.3	1.10, 1.53	0.002
Consult Flag	17,748	326 (2.5%)	539 (11%)	<0.001	3.68	3.14, 4.32	<0.001
Insurance	17,748			<0.001			
Medicaid HMO		4229 (33%)	1299 (27%)		—	—	
Blue Shield		2744 (21%)	977 (20%)		1.11	1.00, 1.23	0.042
Commercial		1278 (9.9%)	395 (8.3%)		0.92	0.81, 1.06	0.3
HAP		503 (3.9%)	223 (4.7%)		1.42	1.19, 1.69	<0.001
Medicaid		989 (7.6%)	493 (10%)		1.45	1.27, 1.65	<0.001
Medicare		695 (5.4%)	405 (8.5%)		1.29	1.09, 1.52	0.003
Medicare Advantage		304 (2.3%)	179 (3.7%)		1.34	1.08, 1.67	0.009
Self-pay		1510 (12%)	419 (8.8%)		0.83	0.72, 0.94	0.004
Tricare		73 (0.6%)	23 (0.5%)		0.98	0.59, 1.56	>0.9
Worker's comp		647 (5.0%)	363 (7.6%)		1.5	1.29, 1.75	<0.001
Level of trauma	17,748			<0.001			
Level I		2296 (18%)	1130 (24%)		—	—	
Level II		2417 (19%)	1141 (24%)		1.29	1.15, 1.46	<0.001
Level III		2820 (22%)	1013 (21%)		1.01	0.90, 1.14	0.8
No trauma designation		5439 (42%)	1492 (31%)		0.83	0.75, 0.93	0.001

CI = confidence interval, HMO = Health Maintenance Organization, OR = odds ratio.

a n (%); mean (SD).

b Pearson chi square test; Wilcoxon rank-sum test; Fisher exact test.

Decreased follow-up in new patients returning for hand trauma–related subsequent care was seen with female (OR 0.6, *P* < 0.001), ‘Arab or Chaldean’ (OR 0.54, *P* < 0.001), Medicaid/Medicaid HMO (OR 0.74, *P* < 0.001), and self-pay (OR 0.63, *P* < 0.001) patients.

### Revenue Generation

For the 9,357 patients (16%) returning for hand trauma–related care, 27,177 encounters were generated, leading to $6.7M in revenue within 2 years of hand trauma presentation (Tables 5 and 6). Potential lost revenue from patients who did not follow-up for injury-related care totaled $34.3M.

**Table 5:** Charges and Collections for all Subsequent Encounters

	Unique Patients	Encounters	Charges (USD)	Net Revenue (USD)
Primary care				
Family Practice	11,262	53,074	27,221,305	9,889,712
Geriatrics	23	58	19,321	8804
Internal medicine	10,793	62,748	80,222,207	25,147,435
Pediatrics	2590	7943	4,191,140	1,580,113
Total primary care	24,668	123,823	111,653,973	36,626,064
Nonsurgical specialties				
Cardiology	3871	18,546	86,194,102	28,892,838
Endocrinology	1075	3481	5,094,233	1,894,777
Gastroenterology	3425	9017	45,129,232	14,088,766
Vascular medicine	0	0	0	0
Infectious disease	1605	4484	34,026,341	13,516,368
Emergency medicine	27,806	117,670	246,188,505	58,263,488
Dermatology	2599	6509	6,600,612	3,079,611
Neurology	2590	6080	26,364,721	8,259,887
Rehabilitation	566	1527	7,429,164	3,462,329
Psychiatry	2583	11,515	30,598,658	13,105,002
Other	7865	44,558	172,468,742	61,119,779
Total nonsurgical specialties	53,985	223,387	660,094,310	205,682,846
Surgical specialties				
Anesthesia	1760	5665	12,319,885	2,760,230
Neurosurgery	1003	3500	35,857,405	11,370,265
Orthopaedics	8418	34,953	96,908,528	34,041,606
Trauma surgery	83	94	1,968,092	574,162
Other	14,319	71,025	254,147,802	84,514,385
Total surgical specialties	25,583	115,237	401,201,712	133,260,647
Overall total	38,720	462,447	1,172,949,995	375,569,558

**Table 6:** Charges and Collections for all Returning for Injury-Related and Injury-Unrelated Services

	Injury-Related Care				Injury-Unrelated Care			
	Unique Patients	Encounters	Charges (USD)	Net Revenue (USD)	Unique Patients	Encounters	Charges (USD)	Net Revenue (USD)
Primary care								
Family practice	1786	2719	1,461,709	411,604	10.919	50,355	25,759,596	9,478,107
Geriatrics	3	4	1137	866	23	54	18,184	7938
Internal medicine	1435	2070	1,670,981	428,271	10,603	60,678	78,551,226	24,719,164
Pediatrics	202	218	54,836	26,363	2549	7725	4,136,304	1,553,751
Primary care total	3426	5011	3,188,663	867,104	24,094	118,812	108,465,310	35,758,959
Nonsurgical specialties								
Cardiology	57	86	2,691,208	143,895	3854	18,460	83,502,894	28,748,944
Endocrinology	12	14	44,168	10,319	1070	3467	5,050,065	1,884,458
Gastroenterology	9	9	36,325	12,182	3421	9008	45,092,907	14,076,585
Vascular medicine	0	0	0	0	0	0	0	0
Infectious disease	127	255	535,244	103,091	1551	4229	33,491,097	13,413,276
Emergency medicine	175	203	708,416	124,416	27,763	117,467	245,480,090	58,139,072
Dermatology	44	72	85,370	41,260	2582	6437	6,515,242	3,038,352
Neurology	135	147	924,604	105,655	2510	5933	25,440,118	8,154,232
Rehabilitation	17	42	45,610	23,321	562	1485	7,383,554	3,439,008
Psychiatry	3	3	708	401	2583	11,512	30,597,950	13,104,601
Other	508	1089	4,705,539	951,580	7708	43,469	167,763,202	60,168,199
Nonsurgical specialty total	1087	1920	9,777,191	1,516,120	53,604	221,467	650,317,118	204,166,726
Surgical specialties								
Anesthesia	136	233	1,040,544	46,667	1704	5432	11,279,341	2,713,562
Neurosurgery	11	17	24,343	5581	1002	3483	35,833,062	11,364,684
Orthopaedics	3988	10,960	5,759,857	2,303,205	6428	23,993	91,148,671	31,738,401
Trauma surgery	1	1	65	0	82	93	1,968,027	574,162
Other	3129	9035	5,239,467	2,004,677	13,037	61,990	248,908,336	82,509,709
Surgical specialty total	7265	20,246	12,064,276	4,360,130	22,253	94,991	389,137,437	128,900,517
Overall total	9357	27,177	25,030,130	6,743,355	37,807	435,270	1,147,919,865	368,826,203

For the 8638 new patients (49%) who received subsequent care, 40,091 encounters were generated. These totaled over $34M in revenue collection within 2 years of presentation for hand trauma over a 4-year period, averaging $8.5M per year; only $980k was because of the hand trauma (Tables 7 and 8). The 9109 new patients lost to follow-up created a potential revenue loss of over $36M.

**Table 7:** Charges and Collections for New Patients’ Subsequent Encounters

	Unique Patients	Encounters	Charges (USD)	Net Revenue (USD)
Primary care				
Family practice	1327	3877	2,418,147	804,732
Geriatrics	1	1	168	116
Internal medicine	951	2586	5,532,466	1,433,582
Pediatrics	428	942	649,379	205,538
Total primary care	2707	7406	8,600,160	2,443,968
Nonsurgical specialties				
Cardiology	249	661	5,718,055	1,616,208
Endocrinology	59	157	199,916	73,405
Gastroenterology	248	590	2,877,086	918,274
Vascular medicine	0	0	0	0
Infectious disease	174	434	2,893,690	1,028,264
Emergency medicine	5857	13,113	26,602,335	6,126,308
Dermatology	217	385	440,717	189,129
Neurology	217	419	2,181,676	649,477
Rehabilitation	59	180	420,284	193,056
Psychiatry	286	996	3,429,493	1,424,743
Other	709	2506	9,620,571	3,428,104
Total nonsurgical specialties	8075	19,441	54,383,822	15,646,968
Surgical specialties				
Anesthesia	157	368	809,459	170,468
Neurosurgery	86	286	3,422,363	1,030,708
Orthopaedics	1590	5371	12,573,339	4,327,946
Trauma surgery	6	6	84,851	23,820
Other	2056	7213	33,558,200	10,681,686
Total surgical specialties	3895	13,244	50,448,212	16,234,630
Overall total	8638	40,091	113,432,194	34,325,566

**Table 8:** Charges and Collections for New Patients Returning for Injury-Related and Injury-Unrelated Services

	Injury-Related Care				Injury-Unrelated Care			
	Unique Patients	Encounters	Charges (USD)	Net Revenue (USD)	Unique Patients	Encounters	Charges (USD)	Net Revenue (USD)
Primary care								
Family practice	191	264	311,000	40,887	1242	3613	2,107,147	763,846
Geriatrics	0	0	0	0	1	1	168	116
Internal medicine	78	103	448,804	25,077	925	2483	5,083,662	1,408,505
Pediatrics	19	21	10,719	2841	415	921	638,660	202,697
Primary care total	288	388	770,523	68,804	2583	7018	7,829,637	2,375,164
Nonsurgical specialties								
Cardiology	5	5	444,606	360	245	656	5,273,448	1,615,848
Endocrinology	0	0	0	0	59	157	199,916	73,405
Gastroenterology	1	1	1201	231	248	589	2,875,885	918,043
Vascular medicine	0	0	0	0	0	0	0	0
Infectious disease	22	45	119,728	4282	158	389	2,773,962	1,023,982
Emergency medicine	32	32	150,038	20,420	5844	13,081	26,452,297	6,105,888
Dermatology	7	11	39,737	19,550	212	374	400,980	169,579
Neurology	11	11	273,306	14,662	208	408	1,908,370	634,815
Rehabilitation	3	20	13,833	10,470	58	160	406,451	182,586
Psychiatry	1	1	236	134	286	995	3,429,257	1,424,609
Other	41	49	108,703	14,764	679	2457	9,511,868	3,413,339
Nonsurgical specialty total	123	175	1,151,388	84,873	7997	19,266	53,232,434	15,562,094
Surgical specialties								
Anesthesia	19	31	18,256	6723	150	337	791,203	163,745
Neurosurgery	1	2	215	178	86	284	3,422,148	1,030,531
Orthopaedics	877	2344	1,114,997	454,430	1053	3027	11,458,342	3,873,517
Trauma surgery	0	0	0	0	6	6	84,851	23,820
Other	797	2411	1,155,688	364,909	1634	4802	32,402,512	10,316,777
Surgical specialty total	1694	4788	2,289,157	826,240	2929	8456	48,159,056	15,408,390
Overall total	1914	5351	4,211,067	979,917	8136	34,740	109,221,127	33,345,648

## Discussion

This study aimed to determine the economic effect of hand trauma and patient loss to follow-up as well as to identify factors that lead to increased continuity of care after hand trauma presentation. Overall, our study found that there is leakage of a notable percentage of patients from a hand trauma program in a large healthcare system (84%); however, despite this leakage, a hand trauma program continues to provide notable revenue. Relative to our primary hypothesis, we found that older, established patients were more likely to follow-up; however, male patients were not. Pertaining to our second hypothesis regarding revenue potential, we found that ‘other surgical services’ was the source of the most revenue generated, not rehabilitation services.

In comparison with the study by Flanagan et al,^9^ our study was also conducted in a metropolitan area of the Midwest, however, at a multicenter healthcare system as opposed to a single center. This resulted in a higher subject population (56,995 hand trauma encounters compared with 440 orthopaedic trauma encounters). In their study, established patients who were non-White and male were more likely to follow-up after their initial encounter. We also found that established patients were more likely to follow-up when compared with new patients. However, our study found that older patients were more likely to follow-up compared with younger patients. This could possibly be b older patients often having an increased number of comorbidities that could affect their likelihood for follow-up. It is important to recognize these demographic factors that cause loss to follow-up because they could be important opportunities of revenue for an orthopaedic department.

Zelle et al^10^ also studied the patient factors associated with loss to follow-up after orthopaedic trauma. Their study included analysis on sex, insurance type, and smoking status to determine the effect of these factors on likelihood to follow-up. Notably, while their study showed 73.3% follow-up, our study showed only a 39.8% follow-up rate within 6 weeks of presentation. Furthermore, the authors found that male sex and being uninsured or having government insurance decreased the likelihood of follow-up. We did also note that follow-up decreased with patients who were uninsured/self-paid; however, our study noted decreased follow-up with female sex. In addition, they did not find significance with worker's compensation while we did see a notable increase in follow-up for those patients using worker's compensation. These differences may be attributed to health care in a different city and a different type of patient population.

Similar to Hasan et al,^8^ we were able to demonstrate the notable revenue that hand surgery generates. In their study, facility and underlying costs were subtracted from the total revenue generated to determine the net revenue from hand trauma and its subsequent care. In our study, we did not include a cost analysis because revenue is more universal, and therefore, we are unable to discuss profitability. Despite this difference in reported calculation methods, both our study and this study showed notable revenue generated. Dissimilar to our study, the authors included only hand surgeries performed by plastic surgeons; this could limit the profitability of the program because it fails to account for the profit seen by orthopaedic surgeons.

Following the revenue generated by the hand trauma service, the highest revenue-producing specialty was ‘other surgical specialties.’ This can likely be attributed to the sharing of hand call by plastic surgery in our healthcare system because injury-related subsequent care after hand trauma seen by plastic surgeons would be completed by plastic surgery as well (classified under ‘other surgical specialties’). This division of the patient inflow allows hand trauma to be profitable to both orthopaedic and plastic departments, but it is unique to the nature of hand trauma and may have been a notable cause of difference when compared with the study by Flanagan et al^9^ in which the highest subsequent revenue generation came from physical medicine and rehabilitation services. This difference could also be because of the multicenter nature of our system and the many private physical/occupational therapy centers in the area. For injury-unrelated subsequent care contributing to ‘other surgical specialties,’ there are two possible underlying causes in our study: (1). Injuries resulting in hand trauma also required other surgeries that would need subsequent care and/or (2). patients with a positive experience with the hand surgery department came back to the same hospital for subsequent surgeries. The third highest revenue-producing specialty was emergency medicine. The possible causes for this are that the patients were being reinjured or were returning to the emergency department for follow-up care instead of clinics or, because of positive experience in our ED during the initial trauma, they returned for subsequent emergency needs.

When we calculated our lost revenue across all systems in the same way done by Flanagan et al, we saw a potential loss of up to $177M. This shows that loss of patients after hand trauma presentation results in notable loss in revenue while also disrupting continuity of care. We subsequently conducted an additional calculation of loss of revenue based on new patients alone. As the hand trauma episode of care represents an opportunity to integrate new patients into the health system, failure to establish subsequent care results in potential loss of revenue from new patients of up to $36M. Therefore, initiatives directed at improving retention rates of new patients would be financially beneficial.

This study has several limitations. First, all participants were recruited from a single system, which may limit the generalizability of our findings. However, our study included 56,995 patients from a multicenter system, which can render our results more generalizable. Second, because data were analyzed retrospectively, there were incomplete data points, and some factors were unable to be included because of incomplete information. Third, revenue generated from hand trauma in an orthopaedic encounter is limited because half of hand trauma presentations follow up with the plastic surgery department because of split call coverage. Fourth, hand trauma data were collected only from those presenting to the emergency department; this does not account for hand trauma that presented first to primary care or family medicine clinics. Fifth, we did not have complete data on patient addresses and distance from the center of care; therefore, some may have been from out of state/region and unable to follow up within this healthcare system. Sixth, patients with more medical comorbidities or increased number of injuries may be more likely to follow up because of an increased number of providers initiating follow-up care; however, we did not evaluate this correlation. This is an opportunity for future studies. Finally, of the 56,995 patients included, 39,247 were already established within the healthcare system, and we were unable to attribute follow-up care specifically to hand trauma; we reduced this limitation by calculating the revenue generation for injury-unrelated care using only new patients who returned to the system after hand trauma presentation.

Overall, this study demonstrates the effect that patient demographics have on patient likelihood for follow-up. Increased follow-up was seen with patient-specific characteristics including increasing age and ‘Hispanic or Latino’; visit-specific characteristics including transfer from an outside health system, hospital admission, and hand surgery consultation; and payment-specific factors including use of worker’s comp and HAP. In addition, hand trauma introduced many patients to the healthcare system who then generated substantial revenue both through hand trauma–related care and subsequent health care unrelated to hand trauma.
